# Preventing DNA over-replication: a Cdk perspective

**DOI:** 10.1186/1747-1028-3-3

**Published:** 2008-01-22

**Authors:** Andrew CG Porter

**Affiliations:** 1 Department of Haematology, Faculty of Medicine, Imperial College London, Du Cane Road, London W12 ONN, UK

## Abstract

The cell cycle is tightly controlled to ensure that replication origins fire only once per cycle and that consecutive S-phases are separated by mitosis. When controls fail, DNA over-replication ensues: individual origins fire more than once per S-phase (re-replication) or consecutive S-phases occur without intervening mitoses (endoreduplication). In yeast the cell cycle is controlled by a single cyclin dependent kinase (Cdk) that prevents origin licensing at times when it promotes origin firing, and that is inactivated, via proteolysis of its partner cyclin, as cells undergo mitosis. A quantitative model describes three levels of Cdk activity: low activity allows licensing, intermediate activity allows firing but prevents licensing, and high activity promotes mitosis. In higher eukaryotes the situation is complicated by the existence of additional proteins (geminin, Cul4-Ddb1^Cdt2^, and Emi1) that control licensing. A current challenge is to understand how these various control mechanisms are co-ordinated and why the degree of redundancy between them is so variable. Here the experimental induction of DNA over-replication is reviewed in the context of the quantitative model of Cdk action. Endoreduplication is viewed as a consequence of procedures that cause Cdk activity to fall below the threshold required to prevent licensing, and re-replication as the result of procedures that increase that threshold value. This may help to explain why over-replication does not necessarily require reduced Cdk activity and how different mechanisms conspire to prevent over-replication. Further work is nevertheless required to determine exactly how losing just one licensing control mechanism often causes over-replication, and why this varies between cell systems.

## Background

### DNA over-replication: the result of uncontrolled origin licensing

The two main hallmarks of the eukaryotic cell cycle are the duplication of the genome (S-phase) and segregation of the two resulting copies into two progeny cells (mitosis). In the interests of genome stability, S-phases must alternate with mitoses and must exactly duplicate the genome. Genome duplication involves DNA replication starting at multiple origins, each of which must therefore initiate ('fire') only once per S-phase, and only if preceded by mitosis. To achieve this an origin licensing system has evolved: origins cannot fire without being licensed, and then cannot be re-licensed without passing through mitosis, during which conditions permissive for licensing but not firing are restored [1-3]. Biochemically, licensing refers to the combined action of various factors (ORC, cdc6, Cdt1, MCM2-7) in assembling a pre-replication complex at replication origins, a process that occurs in G1 prior to origin firing in S-phase.

**Figure 1:**
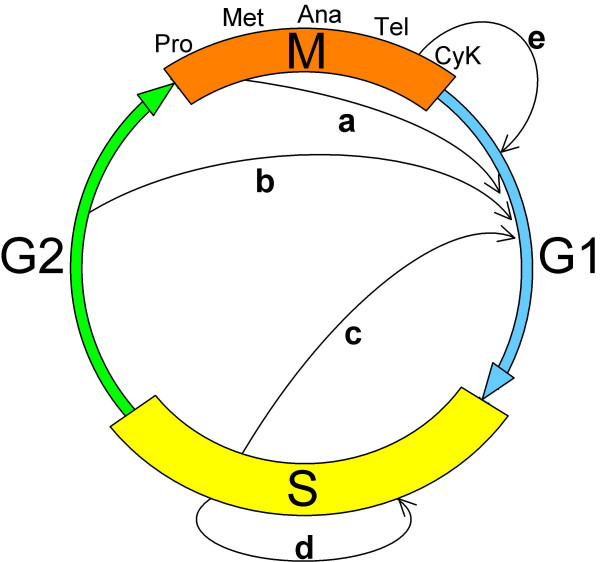
**Pathways of over-replication**. The G1 state required for coordinated origin licensing is normally reached by passing through mitosis. Endoreduplicating cells reach G1 from early M (a), G2 (b) or S (c). Re-replication (d) involves a continuous S-phase during which origin licensing and firing are both permitted. Endomitosis (e) occurs when cytokinesis (CyK) is bypassed but does not involve premature licensing. This figure is similar to Fig. 3 in [5] describing naturally occurring endoreduplication. Pro, Met, Ana, Tel refer to prophase, metaphase, anaphase and telophase, respectively.

Failures in licensing control can give rise to two basic types of DNA over-replication: endoreduplication and re-replication. (The terminology of Arias and Walter [4] is used here, but it should be noted that in many publications, the term re-replication is used more broadly to encompass both re-replication and endoreduplication). In endoreduplication, S-phase is no longer dependent on the passage through mitosis and multiple consecutive S-phases can occur (Fig. 1a,b,c) giving rise to discrete increases in DNA content; within a single S-phase, however, origins still fire only once. Endoreduplication occurs naturally in certain cell types as part of their normal developmental programme [5]. In contrast, re-replicating cells no longer maintain the temporal separation of origin licensing and firing. Thus, during re-replication, origins can fire more than once within a single S-phase leading to a continuous increase in DNA content (Fig. 1d). Re-replication does not appear to occur as part of any natural developmental programme. Experimentally, endoreduplication (at least from G2 or M) and re-replication are usually distinguished by flow cytometric measurements of DNA content, the former giving rise to discrete peaks corresponding to 8 N, 16 N, 32 N etc. (N = haploid chromosome number), the latter generating a range of DNA contents with intermediate values (e.g. between 4 N and 8 N). Endoreduplication from S-phase is also expected to generate intermediate values and so is probably difficult to distinguish from re-replication by this method. Discrete doublings in ploidy do not necessarily indicate endoreduplication; they can also result from failures in cytokinesis (Fig. 1e), but these involve no change in licensing control and will not be considered further here.

### Cdk and APC/C: mutually antagonistic drivers of the cell cycle

The master controllers of the cell cycle are the cyclin dependent kinases [6,7] and the anaphase promotion/cyclosome (APC/C) complex [8,9]. When heterodimerised with their cyclin partners, which are periodically degraded at various points during the cell cycle, Cdks promote both S-phase and mitosis by phosphorylating key target proteins. The single Cdk in fission and budding yeast (Cdc2 and Cdc28 respectively; hereafter Cdk1) associates with multiple cyclins but, at least in fission yeast, a viable cell cycle can be supported by a single cyclin [10]. In budding yeast, cells with deletions in all six B-type cyclin genes [11] or in all three G1-type cyclin genes [12] can be rescued by expression of a single B-type cyclin gene.

In higher eukaryotes multiple Cdks and cyclins exist, the key combinations being Cdk1/cyclin A and B at mitosis, Cdk1 and 2/cyclin A and E during G1/S and Cdk4 and 6/cyclin D during G1 [6]. While different Cdk/cyclin combinations are required in specific lineages during development, accumulating evidence [13-18], particularly the recent generation of viable mouse embryo fibroblasts (MEFs) deleted for Cdk2, Cdk4 and Cdk6 [19], indicates that Cdk1 is the only major Cdk that is absolutely required for viable cell cycles. Furthermore, in frog egg extracts mitotic cyclins are capable of supporting S-phase functions [20,21], and the major cyclin genes can be individually deleted in mice without completely preventing cell proliferation [22]. It has yet to be determined, however, whether higher eukaryotes can, like fission yeast, support viable cell cycles with just a single cyclin/Cdk1 combination.

The counterbalance to Cdk activity is provided by the APC/C, a ubiquitin ligase complex which has many substrates but most crucially targets mitotic cyclins at anaphase for degradation [8,9]. This effectively eliminates Cdk activity, allowing for a period (late M and G1) that is permissive for origin licensing. For the rest of the cell cycle (S, G2 and early M) APC/C must be inactivated, so that mitotic cyclins (A and B) can re-accumulate, and this is achieved (at least in part; see below) by inhibitory, Cdk-mediated phosphorylation of Cdh1, the G1 activating subunit of APC/C. During mitosis, however, high Cdk1 activity *activates *another APC/C activator, Cdc20, leading to the destruction of mitotic cyclins that allows mitotic exit and activation of APC/C^Cdh1^. APC/C and Cdk are thus mutually antagonistic and the cell cycle oscillates between periods dominated by APC/C or Cdk activity.

### DNA over-replication and the quantitative model of Cdk action in yeast

In addition to its roles in promoting S phase and mitosis, Cdk has a third role: preventing DNA over-replication. This role was revealed in fission yeast when a temperature sensitive Cdk1 allele was transiently heat-inactivated after G2 arrest, causing the cells to bypass mitosis, to 'reset' in G1 and undergo a second S-phase [23]. Furthermore, multiple consecutive S-phases could be induced by deletion of Cdc13 [24], which encodes the mitotic cyclin partner of Cdk1, or by over-expression or Rum1 [25,26], an inhibitor of Cdk1 in G2. Such endoreduplication was demonstrated by discrete doublings in the genome size. Similar results were obtained in budding yeast [27].

**Figure 2:**
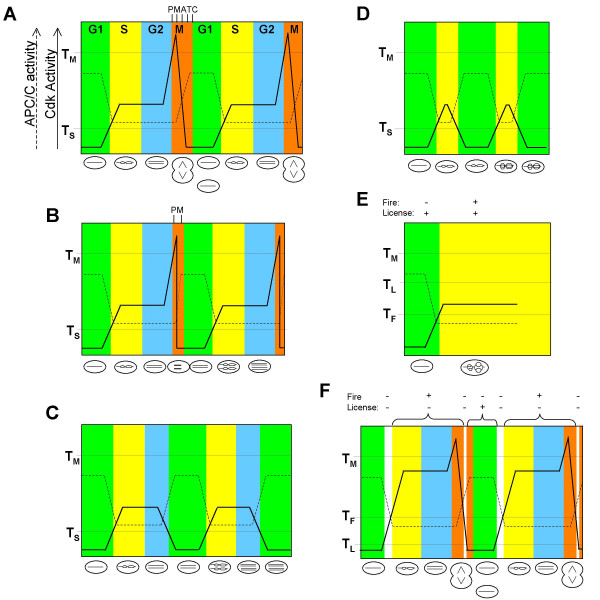
**Quantitative model of Cdk activity applied to normal and over-replicating cell cycles**. Cdk (continuous line) and APC/C (broken line) activities (y-axis) are plotted against time (x-axis). T_M_, T_S_, T_L _and T_F _indicate thresholds of Cdk activity, as defined in the text. P,M,A,T and C: prometaphase, metaphase, anaphase telophase and cytokinesis, respectively. A. Normal cycle. B. Endoreduplication from mitosis. C. Endoreduplication from G2. D. Endoreduplication from S-phase. E. Re-replication. F. Normal cell cycle with periods of insulation (white segments) when origins can neither be licensed nor fire. Fire +/- = sufficient/insufficient Cdk to promote origin firing. License +/- = sufficient/insufficient Cdk to prevent licensing.

The problem of how a single Cdk could suppress over-replication yet also promote S-phase and M-phase was addressed in the quantitative model of Cdk action [10,28]. It was proposed that, following exit from mitosis, rising levels of Cdk activity pass through two thresholds. Below the first threshold (T_S_), in G1, replication origins can be licensed but cannot fire, but above T_S _origins can fire but cannot be re-licensed. Thus transition above T_S _marks the beginning of S-phase and ensures that origins fire only once per S-phase. The second threshold (T_M_) is passed when inhibitory phosphorylation sites on Cdk1 are removed by Cdk1-activated Cdc25 phosphatase, resulting in a highly activated Cdk1 that promotes the onset of mitosis [29]. Destruction of mitotic cyclins by the APC/C allows mitotic exit and completes the cycle (Fig. 2A).

Although the quantitative model is strongly supported by the observation that a single cyclin/Cdk1 complex can guide the cell cycle of fission yeast [10], a degree of qualitative control is also evident. Thus, individual cyclin/Cdk combinations are known to have specific functions [3,30,31] and these are likely to contribute to the fine tuning of cell cycle control, especially in higher vertebrates.

Endoreduplication is explained in the context of the quantitative model as follows (Fig. 2B,C). In G2, or early M, experimental depletion of Cdk activity causes premature activation of APC/C^Cdh1^, without the need to traverse mitosis and activate APC/C^Cdc20^. The cells therefore reset in G1, co-ordinately relicense all origins and, when Cdk is reactivated, enter the next S-phase. For multiple endocycles, the loss of Cdk activity must be repeated each time the cells reach G2/M. This can be achieved experimentally, (e.g. by repeated temperature shifts in tsCdk1 mutants) or spontaneously, by residual cell cycle oscillations. Thus, multiple endocycles seen after Cdc13 deletion in fission yeast reflect the periodic synthesis and inactivation of the S-phase cyclins. In principle, those cells in S-phase at the time of Cdk1 depletion should also reset in G1 (Fig 2D), in which case restoration of Cdk1 activity would lead to duplication of a partially replicated genome, i.e. discrete but variable increases in ploidy to less than 8 N. This form of endoreduplication does not appear to have been described after the experimental depletion of Cdk1 activity, possibly because of difficulties in distinguishing it on the basis of DNA content from other forms of over-replication, but it does occur naturally in Drosophila larval tissue [5].

To describe re-replication in terms of the quantitative model it is important to remember that, by definition, it involves conditions that simultaneously permit both origin licensing and firing. Multiple overlapping mechanisms by which Cdk1 can prevent origin licensing in yeast have been revealed. These include inhibition of ORC, nuclear export of Cdt1 and MCM2-7, and inactivation, degradation and reduced synthesis of Cdc6 ([32]; see [4] for a recent review). More recently the essential Cdk1 substrates (Sld2, Sld3) whose phosphorylation leads to origin firing have been identified [33]. These studies do not appear to indicate that the threshold Cdk activities for inhibiting origin licensing (T_L_) and for promoting origin firing (T_F_) need necessarily coincide. The possibility therefore exists that these are independent thresholds and that, under normal circumstances, parameters are set such that T_F _> T_L_. In this model, re-replication is caused by any procedure that disturbs such parameters to cause T_F _< T_L _(Fig. 2E). In budding yeast, multiple components of the preRC complex are sensitive to Cdk1 phosphorylation and so conspire to keep T_L _low, but when enough of the relevant phosphorylation sites are mutated, re-replication follows [32]. Alternatively, modifications to Sld2 and Sld3 that remove their dependence on phosphorylation by Cdk1, effectively reduce the value of T_F _and so cause re-replication [33].

This proposed separation of T_S _into separate thresholds (T_F _and T_L_) has the additional attraction that it predicts an 'insulation' period at the G1/S transition, and also at anaphase, during which origins can neither be licensed nor fire, ensuring that there is no overlap between periods of origin licensing and firing (Fig. 2F). Although insulation in budding yeast has been elegantly explained in terms of different cyclin specificities [3], this quantitative model is potentially more appropriate in fission yeast, at least, where viable cycles require only a single Cdk/cyclin combination.

How far can these descriptions of endoreduplication and re-replication in yeast be applied in mammalian cells?

### Endoreduplication from G2 or M after Cdk1 inactivation in mammalian cells

By analogy with the work in yeast, experimental depletion of Cdk1 in higher eukaryotes might be expected to promote endoreduplication and this is indeed the case in some cell lines. Thus endoreduplication was seen in human fibrosarcoma cells (HT2-19) with repressible Cdk1 gene expression [34] and in human osteosarcoma (U2OS) cells depleted of Cdk1 by RNA interference [35]. Because Cdk1 depletion was sustainable in these studies, it was possible to detect multiple endocycles, generating ploidies as high as 64 N. (In contrast to yeast, multiple endocycles do not require multiple transient Cdk1 inactivations because S-phases do not require Cdk1.) These endocycles apparently correspond to endoreduplication from G2 (Fig. 2C), bypassing mitosis but maintaining normal S-phases. Thus endoreduplicating HT2-19 cells do not undergo nuclear envelope breakdown or activate the APC/C^Cdc20^, but retain cycles of cyclin E expression and centrosome duplication [36]. Also consistent with the model in Fig. 2C is the ability of Cdk1 that lacks inhibitory phosphorylation sites, and is therefore unresponsive to Cdc25 phosphatase, to prevent endoreduplication in HT2-19 without promoting mitosis [37].

Endoreduplication is sometimes seen after DNA damage and this may also be explained in terms of reduced Cdk1 activity [38]. Although G2 checkpoint pathways are known to prevent Cdk1 activation by preventing the removal of inhibitory phosphorylations [39], this is only predicted to prevent Cdk1 levels from exceeding the T_M _threshold, and so to prevent mitosis. However, sufficiently intense or sustained levels of DNA damage can cause transcriptional silencing of various genes expressed in S/G2, including Cdk1 [38,40], and this more easily explains DNA damage-induced endoreduplication. Cdt1 is also degraded in response to DNA damage by the Cdk-independent Cul4-Ddb1^Cdt2 ^pathway ([4];see below) and this effect is presumably lost in cells that over-replicate in response to DNA damage.

Endoreduplication has also been observed (in SaOS cells) after indirectly depleting Cdk1 activity with high levels of the Cdk inhibitors p21 or p27 [41,42] or (in HCT-116 cells) with siRNA to cyclin B [43]. Endoreduplication in response to Cdk inhibitor over-expression was found to occur in cells (SaOS, HeLa and in Rb-/-MEFs) lacking functional Retinoblastoma protein (Rb) but not in control cells (Rb+/-MEFs, RKO, Rat1, A549, H1299, HaCat, SW480) with a functional Rb [41]. This is expected if endoreduplicating cells reset in G1 because Cdk inhibition at this stage would prevent the Rb phosphorylation required to release the E2F transcription factor necessary for the G1/S transition. Cyclin B-depleted HCT116 cells still show signs of early mitosis, presumably due to Cdk1/cyclin A activity, but they exit mitosis before metaphase [43].

In some studies, involving NCI-H1299 [35] or HeLa and HePG2 cells [44], appreciable endoreduplication required depletion of both cyclin A and B, or both Cdk1 and Cdk2. There is evidence that Cdk2/cyclinA can promote Cdk1 activation [45] and this may help to explain the enhanced phenotype after cyclin A depletion. It is less easy to explain how depleting Cdk2 activity, including Cdk2/cyclinA, can promote endoreduplication when it is essential for origin firing in the absence of Cdk1. It is possible, however, that Cdk4 or Cdk6 can substitute for Cdk2 at G1/S, as Cdk1 is capable of doing [14,46]. Alternatively, depletion of cyclin A or Cdk2 in these studies may have been more effective at inactivating Cdk1 than Cdk2.

It is notable than in none of the above studies was there any clear evidence of re-replication i.e. of partial rather than discrete increases in ploidy. This is consistent with the models of Fig. 2 because changes in Cdk activity are not predicted to alter the values T_F _and T_L_. However, partial increases in ploidy might have been expected if endoreduplication had been induced from S-phase, suggesting that other pathways prevent endoreduplication during S-phase.

There are several mechanisms by which Cdk activity may inhibit origin licensing (reviewed in [4]). The clearest seems to be degradation of Cdt1 by the SCF^Skp2 ^pathway in response to its phosphorylation by Cdk, particularly in G2 [47,48]. Also there are data to suggest that the binding of Cdt1 [49,50], ORC and cdc6 [51-54] may each be subject to control by Cdk activity. If sufficient Cdk target sites in the licensing machinery can be mutated, this would be expected to increase the value of T_L _and induce re-replication, but only if Cdk-independent mechanisms of inhibiting licensing are also inactivated (see below). The importance of Cdt1 and Cdc6 as targets for the control of re-replication was demonstrated by the re-replication seen when Cdt1 and Cdc6 were over-expressed in H1299 cells [55].

### Cells that do not over-replicate after Cdk1 inactivation

Contrasting with the above studies are reports of cell lines where Cdk1 depletion does not result in over-replication. Thus Cdk1 depletion by siRNA failed to induce over-replication in NCI-H1299 cells [35], although as already mentioned, co-depletion of Cdk2 induced endoreduplication. FT210 cells, whose endogenous Cdk1 protein is temperature-sensitive, arrested in G2 at non-permissive temperature; no over-replication was seen 20 h after temperature-shift [56], but it would be interesting to know the situation at later times. Similarly, chemical inhibition of Cdk1 in asynchronous cultures of HeLa, SW480 or HCT116 cells for 20 h [57], or DT40 cells for 48 h [18], caused only a G2 arrest. In all these cell lines, however, including FT210, Cdk1 inactivation after presynchronisation in mitosis by treatment with nocodazole, did lead to endoreduplication. Thus, in these cell lines, Cdk1 appears to be alone in suppressing endoreduplication during early mitosis, but not in G2. A formal, and potentially interesting explanation for why endoreduplication from G2 is seen after Cdk1 depletion but not after chemical inactivation, would arise if a kinase-independent property of Cdk1 were capable of suppressing endoreduplication in G2. The results in FT210 cells, where Cdk1 is degraded at the non-permissive temperature, argue against this, and a less radical explanation involving Emi1 has recently emerged (see below). Nevertheless, a direct comparison in the same cells of the effects of Cdk1 depletion and inhibition would be valuable. Whatever the outcome, these results indicate that, in several cell lines, Cdk1 activity is not alone in being able to suppress licensing in interphase, a conclusion that has been reached by several other lines of investigation.

### Geminin and Cul4-Ddb1^Cdt2 ^suppress over-replication by regulating Cdt1

Geminin is a protein present in metazoans, but not in yeast, that suppresses origin licensing through its ability to bind and inactivate Cdt1 [58,59]. Geminin is stable during S, G2 and early M, but targeted for degradation by APC/C and therefore degraded during mitosis, allowing for origin licensing until it is resynthesised in late G1. Two pathways target Cdt1 for degradation. The Cul4-Ddb1^Cdt2 ^pathway requires Cdt1 to interact with PCNA and therefore operates only in S-phase, but is apparently independent of Cdk activity. This contrasts with Cdt1 degradation by SCF^Skp2^, which operates mainly in S/G2/M and, as mentioned above, depends on phosphorylation of Cdt1 by Cdk1. By reducing the amount of free Cdt1, these mechanisms both effectively reduce the level of Cdk activity required to suppress licensing, i.e. reduce the value of T_L_, during S, G2 and early mitosis. Initially these mechanisms may have evolved to further reduce the chances of premature re-licensing and to improve the 'insulation' during transitions between phases of high Cdk1 activity and high APC/C activity. Over time, however, mammalian cells have apparently become dependent on them to ensure that T_L_< T_F_. Thus, depletion of geminin (in S/G2/M) or Cdt2 (in S) may be sufficient to raise the value of T_L _above that for T_F _and so cause re-replication. Accordingly, depletion of geminin in HCT116, U2OS and TIG3 cells [60-62], and Cdt2 [63]or Ddb1 [64] in HeLa cells, all cause over-replication characterised by the partial increases in ploidy expected for re-replication. Geminin depletion in HeLa and MCF10A cells does not cause over-replication [60,65,66], however, suggesting that the Cul4-Ddb1^Cdt2 ^pathway plays the predominant role in these cells during S phase. In mouse embryos, geminin down-regulation is associated with endoreduplication and the formation of polyploid cells of the trophoblast lineage [67], rather than with re-replication, suggesting that other pathways prevent licensing during S-phase.

Interestingly, over-replication (apparently involving a mix of partial and discrete increases in ploidy) is seen after geminin depletion in HeLa cells if cyclin A is also depleted (cyclin A depletion alone causing very modest endoreduplication)[60]. It is also notable that in cases where over-replication was caused by geminin or Cdt2 depletion it was less pronounced when DNA damage-induced checkpoints that lead to Cdk1 inactivation were blocked with caffeine [61,62]. A possible explanation for these observations is that in some cells, even when T_L_> T_F_, Cdk activity must be limited so that it stays between these two threshold values in order for appreciable re-replication to occur. The question remains, however, of why the Cul4-Ddb1^Cdt2 ^pathway in HeLa cells, whose inhibition is sufficient to cause re-replication [63,64], does not prevent re-replication after the combined depletion of geminin and cyclin A[60]. The possibility should be considered, therefore, that the over-replication caused by depleting both geminin and cyclin A is primarily endoreduplication and that apparent partial increases in ploidy reflect a secondary phenomenon (e.g. apoptosis) and not re-replication.

While the roles of geminin and the Cul4-Ddb1^Cdt2 ^pathway in suppressing licensing clearly overlap with the role of Cdk1, there are difficulties in explaining how they could be responsible for suppressing origin licensing in those cases where Cdk1 depletion does not cause endoreduplication. This is because Cdk1 depletion is expected to result in APC/C activation, at least in G2/M, and therefore geminin degradation, while the Cul4-Ddb1^Cdt2 ^pathway only operates in S-phase. The solution to this problem appears to lie with Emi1.

### Emi1 suppresses over-replication by inhibiting APC/C

Just as the role of Cdk in suppressing licensing is supported by the actions of geminin and the Cul4-Ddb1^Cdt2 ^pathway, so its role in suppressing APC/C during S and G2 is assisted by Emi1. Emi1 is an APC/C inhibitor that interacts with Cdc20 [68] and Cdh1 [69,70]. It is synthesised when the transcription factor E2F is activated at the G1/S boundary [69]and degraded by SCF-dependent proteolysis early in mitosis promoted by Plk1[71]. Emi1 therefore keeps APC/C^Cdh1 ^inactive during S and G2, allows mitotic cyclins and geminin to accumulate. In two recent studies where Emi1 was depleted by siRNA in MCF10A, HCT116 and HeLa cells, over-replication was observed, accompanied by reduced levels of cyclins A and B1 and geminin [60,72].

This newly established role for Emi1 may help to explain the different responses between cell lines to Cdk1 inactivation. In some cell lines Emi1 may be sufficiently abundant or available to prevent APC/C activation during S/G2, even when Cdk1 is inactive. For example, it was only in mitosis, when Emi1 is degraded, that Cdk1 inhibition caused endoreduplication in various cell lines [18,57]. In G2, despite Cdk1 inactivation, geminin was stable and APC/C remained inactive [18]. In others cell lines, such as HT1080, however, Emi1 presumably plays a less decisive role than Cdk1 in preventing APC/C activation because Cdk1 depletion causes endoreduplication from G2 [34,35]. Further work is required to test the validity of this explanation and to determine the basis of any differences in Emi1 function between cell lines.

The over-replication seen after Emi1 depletion in HeLa and HCT116 cells appears mainly to involve partial increases in ploidy, suggestive of re-replication [60,72]. This is somewhat puzzling because activation of APC/C following Emi1 depletion is expected to reset cells in a G1-like state and so cause endoreduplication. Also, in HeLa cells at least, the Cul4-Ddb1^Cdt2 ^pathway should be capable of preventing origin licensing in S-phase, and therefore re-replication. The phenotype in Emi1-depleted MCF10A is more suggestive of endoreduplication [60], however, and it will be interesting to characterise in more detail, and in more cells lines (including non-transformed cells), the nature of the over-replication caused by Emi1 depletion.

### Overview of over-replication control

**Figure 3:**
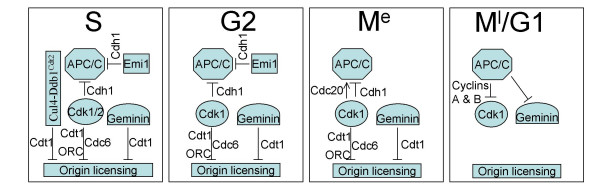
**Key regulators of over-replication higher eukaryotes**. The relationships between Cdk, APC/C, geminin, Cul4-Ddb1^Cdt2 ^and Emi1 during different cell cycle phases are summarised. M^e ^and M^l^, early and late mitosis, respectively.

The key factors that regulate origin licensing and therefore DNA over-replication at various stages of the vertebrate cell cycle are summarised in Fig. 3. The central role of Cdk is evident, involving not only its ability to inhibit APC/Cdh1 but also the licensing machinery. It can also be seen how APC/C (and therefore Emi1) is crucial in licensing control because of its role in depleting both geminin and, via cyclin degradation, Cdk1/2 activity [73]. Thus we can see how depleting Cdk activity in mitosis (and in G2 if Emi1 is inactive), or promoting APC/C^Cdh1 ^activity, can cause endoreduplication by reprogramming cells into a G1-like state without the need to pass through mitosis.

Less clear from this summary is how altering just one of the multiple mechanisms for licensing inhibition so often leads to re-replication, and how this can differ between cell lines. The simplest explanation is perhaps that different inhibitory mechanisms contribute unequally and that the balance of contributions is 'set' differently in different cell lines. The example has already been made of how variations in Emi1 availability might explain different responses to Cdk1 inhibition. In another example, we may explain the re-replication caused by geminin depletion in HCT116 cells [60-62] by assuming that Cdk and the Cul4-Ddb1^Cdt2 ^pathway play relatively minor roles, at least in S-phase. The quantitative model of Cdk action can be a useful way of viewing this: geminin is essential to ensure T_L_<T_F _in such cells, whereas the Cul4-Ddb1^Cdt2 ^pathway is not. Conversely, in HeLa cells, where depletion of Cul4-Ddb1^Cdt2 ^pathway causes re-replication [63,64], the Cul4-Ddb1^Cdt2 ^pathway is essential to ensure T_L_<T_F_, whereas geminin is not.

Such explanations need to be tested in detail, but even if they are true, difficult questions remain. Why, for example, do HeLa cells appear to re-replicate when both geminin and cyclin A are depleted [60] when other experiments [63,64] indicate that the Cul4-Ddb1^Cdt2 ^pathway in HeLa cells should prevent this? Also, why is re-replication seen after depletion of Emi1 or of geminin and cyclin A [60,72], when these treatments are expected to reset cells in a G1-like state and so cause endoreduplication, as seen after Cdk depletion [34,35,41,42]? There are also technical issues concerning the methods for inactivating licensing control mechanisms and for measuring responses. Is it possible, for instance, that the transient and often incomplete nature of siRNA mediated depletion could generate some misleading phenotypes, and how reliable is flow cytometry as a method for distinguishing endoreduplication and re-replication?

Clearly the scheme in Fig. 3 is just a working model and further mechanisms, and/or links between them, are likely to emerge. In fact cross-talk between mechanisms has been proposed as way to ensure that disruption of just one mechanism causes massive re-replication rather than partial re-replication; the former would lead to apoptosis, the latter to viable cells with genome damage that may eventually threaten the whole organism [4]. To explore such ideas, future work will require detailed comparisons, both within a single cell system and between different cell systems, of the effects of inhibiting each of the mechanisms identified to date.

## Conclusion

The quantitative model of Cdk action remains a useful starting point for considering how DNA over-replication is prevented, even in higher eukaryotes. Thus endoreduplication may be caused by procedures (e.g. Cdk inhibition or loss of APC/C inhibition) that flip the balance of Cdk and APC/C activities from a Cdk^hi^/APC^lo ^state to a Cdk^lo^/APC^hi^, G1-like state. In some systems this balance may be delicately set, such that a single perturbation is sufficient to promote endoreduplication, in others a combination of perturbations may be required. Similarly, re-replication may be caused by procedures that raise the threshold of Cdk activity required to prevent origin licensing above that required to promote origin firing. The balance of mechanisms that determine these thresholds values is also likely to vary between cell systems such that loss of one mechanism may be sufficient to cause re-replication in one system, whereas in other systems more that one mechanism must be impaired. While these explanations are consistent with many features of experimentally induced over-replication, several inconsistencies remain and these explanations will remain speculative until more information, qualitative and quantitative, is obtained on the various mechanisms that control origin licensing.

### Cell lines

Cells referred to in this article (human, unless stated otherwise): A549 (lung carcinoma), DT40 (Chicken B-cell), FT210 (mouse mammary carcinoma with tsCdk1), HaCat (keratinocyte), HCT116 (colon carcinoma), HeLa (cervical carconoma), Hep3B (liver carcinoma), HT1080 (fibrosarcoma), HT2-19 (HT1080 with IPTG-regulated Cdk1), MEFs (mouse embryo fibroblasts), NCI-H1299 (non-small cell lung cancer), MCF10A (immortalised breast epithelial), Rat1 (rat 3T3-like fibroblasts), RKO (colon carconoma), SaOS (osteosarcoma), SW480 (colon adenocarcinoma), TIG3 (normal diploid fibroblasts), U2OS (osteosarcoma).

## Abbreviations

APC/C: anaphase promotion complex/cyclosome; Cdk: cyclin dependent kinase

## Competing interests

The author(s) declare that they have no competing interests.
